# From Athletic Performance to Functional Ageing: Shared Genetic Architecture, Redox-Inflammatory Pathways and Functional Reserve Across the Life Course—A Narrative Review

**DOI:** 10.3390/biomedicines14081705

**Published:** 2026-07-29

**Authors:** Samuel Fernández-Lorenzo, Cristian Marín-Pagán, Lorena Ponce, Juan Gambini, Remus Iulian Lupu, Francisco Javier Martínez-Noguera, Javier Escobar

**Affiliations:** 1 Sabartech S.L., Science Park of the University of Valencia, Carrer del Catedràtic Agustín Escardino Benlloch 9, 46980 Paterna, Spain; samuel.fernandez@sabartech.com (S.F.-L.); lorena.ponce@sabartech.com (L.P.); 2 High Performance Sports Research Centre, Universidad Católica San Antonio de Murcia, 30107 Murcia, Spain; cmarin@ucam.edu; 3 Department of Physiology, Faculty of Medicine and Dentistry, University of Valencia, Av. de Blasco Ibáñez, 15, 46010 Valencia, Spain; juan.gambini@uv.es (J.G.); remustafad@gmail.com (R.I.L.); 4 Overgenes S.L., Science Park of the University of Valencia, Carrer del Catedràtic Agustín Escardino Benlloch 9, 46980 Paterna, Spain

**Keywords:** sarcopenia, frailty, athletic performance, genetics, pleiotropy, functional reserve, gut microbiota, ageing, redox signalling, inflammageing

## Abstract

Physical performance can be understood as a continuum throughout the life course, ranging from peak athletic ability in early life to the preservation of mobility and functional independence in old age. This narrative review explores whether the biological and genetic pathways involved in athletic performance might also modulate the risk of geriatric motor dysfunctions (GMDs), a conceptual umbrella proposed here for sarcopenia, dynapenia, lower-limb weakness and the motor component of physical frailty. The available evidence suggests a convergence between performance and motor decline in mechanisms such as mitochondrial function and mitophagy, anabolic–catabolic balance, oxidative stress and low-grade chronic inflammation, neuromuscular integrity, satellite cell function, mechanotransduction, myokine-mediated signalling, and the gut–muscle axis. Although classic candidate genes such as *ACTN3*, *ACE* and *PPARGC1A* have been useful for formulating mechanistic hypotheses, genome-wide association studies support a highly polygenic architecture for strength, lean mass, muscle weakness and frailty. These effects are strongly modulated by the exposome, particularly by physical activity, nutrition and comorbidities. Overall, the relationship appears consistent with predominantly beneficial pleiotropy, although context-dependent effects cannot be ruled out. Genetics may influence functional reserve and decline trajectories, but exercise, particularly strength and power training, along with adequate nutrition and the management of comorbidities, remain the primary strategies for preventing or delaying sarcopenia, frailty and lower-limb weakness.

## 1. Introduction: From Elite Performance to Functional Independence Across the Life Course

Physical performance exists along a continuum ranging from peak physical capabilities in youth to the maintenance of functional independence in old age. This transition reflects not only inevitable age-related changes, but also complex interactions between genetic factors, environmental exposures and lifestyle factors that shape musculoskeletal function throughout the life course. Over recent decades, biomedical and sports science research has shown that the biological systems that determine athletic performance (cardiorespiratory capacity, energy metabolism, muscle strength and power, etc.) are essentially the same critical systems that aid in the preservation of mobility, the prevention of falls and the maintenance of independence in older adults [1,2]. Physical exercise is therefore relevant not only for improving athletic performance and physical fitness, but also as one of the principal strategies for maintaining health and function during ageing [3]. Data from studies on longevity in elite athletes further support the existence of a biological link between athletic performance and healthy ageing. A comprehensive systematic review involving more than 465,000 elite athletes revealed that former athletes, particularly those who participated in endurance and mixed sports, tend to have lower mortality rates and greater longevity compared to the general population [4]. For example, it has been shown that runners capable of completing a mile in under four minutes live approximately five years longer than the general population [5]. These findings suggest that the physiological systems underlying high physical performance may also contribute to functional resilience and long-term survival.

**Figure 1:**
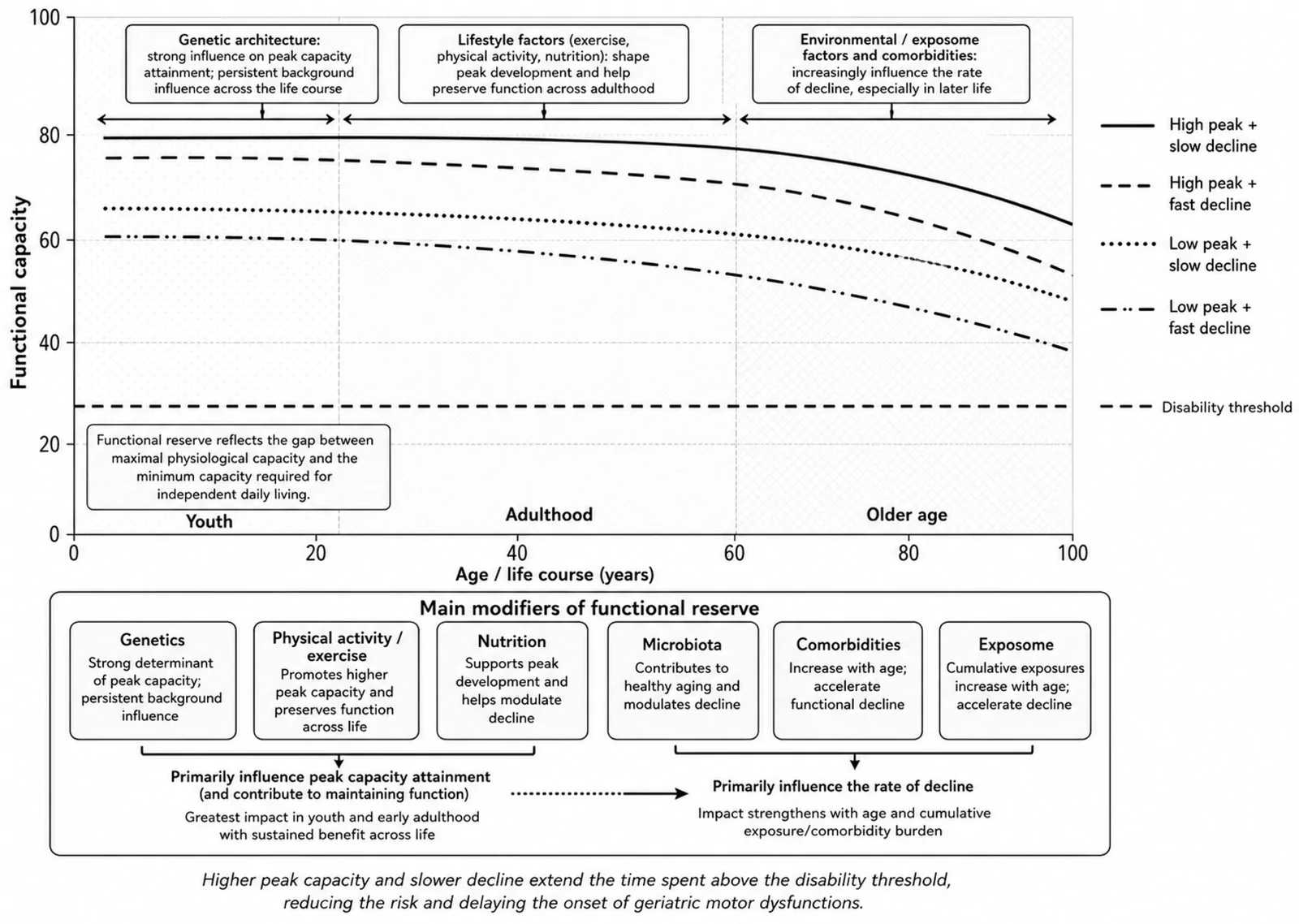
Life-course model of functional reserve. Functional capacity is represented as a trajectory determined by the peak physiological capacity reached in youth or early adulthood and the subsequent rate of decline with ageing. Individuals with a higher functional peak and a slower decline remain above the disability threshold for longer, thereby delaying or reducing the risk of geriatric motor dysfunctions. The upper callouts indicate periods during which different determinants may exert a particularly prominent influence: genetic architecture contributes substantially to the attainment of peak capacity; physical activity, exercise and nutrition shape peak development and help to preserve function during adulthood; and accumulated environmental exposures, comorbidities and other exposome-related factors increasingly influence the rate of decline in later life. The lower panel distinguishes modifiers that predominantly influence peak-capacity attainment from those that increasingly affect the rate of functional decline, while recognising that these influences overlap and interact throughout the life course.

The concept of functional reserve, analogous to cognitive reserve in neuroscience, provides a unifying framework for understanding this continuum. Moreover, functional reserve should be interpreted as the physiological capacity to tolerate stressors while maintaining functional independence. This concept overlaps partially with intrinsic capacity and physical resilience, although it emphasises the dynamic reserve available to preserve mobility and autonomy under conditions of ageing, disease or inactivity [6,7]. Put simply, functional reserve refers to the difference between an individual’s maximum physiological capacity and the minimum threshold required to carry out activities of daily living [8,9]. People who reach higher peaks of aerobic capacity and muscle strength in early adulthood have greater functional reserve, meaning they can lose more absolute function before crossing the threshold into disability [10,11]. This perspective suggests that the peak functional capacity achieved in youth and the subsequent rate of decline are critical determinants of the development of age-related motor dysfunction, such as sarcopenia, frailty or lower-limb weakness, conditions that we here propose to group under the umbrella term “geriatric motor dysfunctions” (GMDs) (Figure 1). To our knowledge, no consensus term encompasses these overlapping motor phenotypes; we therefore introduce GMDs ex novo as a conceptual synthesis for this review.

Furthermore, there is longitudinal evidence to support this framework. In a Swedish cohort of approximately 1.14 million men, higher handgrip strength measured between the ages of 16 and 19 was associated with a substantially lower rate of premature mortality (before the age of 55), even after adjusting for body mass index and blood pressure [12]. Similarly, the CARDIA study showed that higher cardiorespiratory fitness in young adulthood and its maintenance into middle age were associated with a more favourable cardiovascular risk profile [13]. Additional evidence from elite athletic populations further reinforces this perspective, with former elite athletes and individuals capable of exceptional endurance performances exhibiting greater longevity compared with the general population. Although these observational findings may partly reflect selection effects and favourable social, behavioural or biological characteristics, they suggest that physical capacity in early life may reflect not only athletic potential but also broader physiological robustness and resilience into later life.

Importantly, the biological systems underlying athletic performance and healthy ageing appear to overlap substantially [14,15]. Pathways regulating mitochondrial function, neuromuscular integrity, anabolic signalling, mechanotransduction, metabolic flexibility, redox homeostasis and chronic low-grade inflammation are involved both in exercise adaptation and in the preservation of mobility and independence during ageing. Within this network, oxidative stress and inflammation should be considered interdependent processes: transient redox-inflammatory signals contribute to tissue repair and training adaptation, whereas chronic activation promotes tissue dysfunction and progressive functional decline [16,17,18,19,20,21,22,23,24]. However, despite growing evidence linking exercise-related phenotypes with ageing trajectories, these domains have traditionally been studied separately, limiting our understanding of the shared mechanisms that may connect peak physical performance with late-life functional outcomes.

There are therefore several unresolved issues that require a more integrated approach in this regard. First, although candidate-gene studies and GWAS have separately mapped variants influencing athletic phenotypes and late-life motor decline, the extent to which these two genetic architectures overlap remains poorly quantified. Secondly, the molecular and cellular pathways activated by adaptation to exercise and those altered in sarcopenia and frailty are described in publications which, to a large extent, do not overlap, making it difficult to consider the possibility that they represent opposite states of a single underlying network. Third, the relative contributions of favourable and antagonistic pleiotropy to lifespan trajectories of muscle function have not been systematically discussed in the context of GMDs. Finally, preliminary evidence, including the Mendelian-randomisation causal link between grip strength and fracture risk reported by Willems et al. [25], the causal effect of muscle weakness on frailty demonstrated by Jones et al. [26], and the epidemiological longevity gains observed in elite endurance athletes [4,5], is compatible with the hypothesis of a shared biological architecture, but a coherent integrative framework is still lacking.

Therefore, this narrative review aims to synthesise the current evidence on the biological and genetic overlap between sports performance phenotypes and geriatric motor dysfunctions. Specifically, this review focuses on the role of functional reserve, pleiotropy, polygenic influences and life-course trajectories in shaping musculoskeletal function, physical resilience and functional ageing. In doing so, we propose a conceptual framework in which athletic performance and geriatric motor dysfunctions may represent distinct phenotypic manifestations of common biological systems operating across the lifespan.

The scope of this review is deliberately restricted to skeletal muscle-centred motor function and its genetic–environmental modulators across the life course. Four interconnected concepts organise the narrative: (i) functional reserve, understood as the physiological headroom between maximal capacity and the disability threshold; (ii) pleiotropy, both favourable and antagonistic, as the theoretical bridge linking early performance and late-life decline; (iii) polygenic architecture, encompassing candidate genes, GWAS-identified loci and polygenic risk scores (PRS); and (iv) life-course trajectories, integrating early peak, mid-life maintenance and late-life decline. These four concepts are not independent: functional reserve is the phenotypic outcome; pleiotropy is the evolutionary mechanistic hypothesis; polygenic architecture is the biological substrate; and life-course trajectories are the temporal axis on which the previous three concepts operate. Topics not central to this axis, such as neurodegenerative motor disorders (Parkinson’s disease, ALS), cognitive decline or purely cardiovascular ageing, are considered only where they intersect directly with musculoskeletal function.

## 2. Literature Review and Selection Strategy

This review has been designed as a narrative and integrative review. The literature was identified through iterative searches in PubMed, Web of Science and Scopus, with the last search conducted on 20 May 2026. To enhance transparency and reproducibility, the search strategy was structured into four conceptual blocks: (i) physical performance phenotypes (e.g., muscle strength, power output, gait speed, VO_2max_, athletic performance), (ii) age-related functional decline phenotypes (e.g., sarcopenia, frailty, dynapenia, defined as the age-related loss of muscle strength that occurs independently of muscle mass loss, lower-limb weakness), (iii) biological mechanisms (e.g., mitochondrial function, mitophagy, oxidative stress, redox signalling, antioxidant responses, inflammageing, redox-inflammatory pathways, anabolic signalling, neuromuscular integrity, mechanotransduction and metabolic flexibility), and (iv) genetic and epigenetic determinants (e.g., GWAS, polygenic scores, *ACE*, *ACTN3*, *PPARGC1A*, exercise genomics, epigenetic memory and redox-sensitive regulatory pathways). These blocks were combined using Boolean operators (AND/OR) to maximise sensitivity while preserving conceptual specificity. No time restrictions were applied, although priority was given to publications from the last decade, along with studies relevant to the conceptual development of the field. Preference was given to articles published in English, international consensus documents, genome-wide association studies, meta-analyses of large biobanks, randomised clinical trials, longitudinal studies and high-impact mechanistic reviews.

The selection of references was based on their conceptual relevance, methodological rigour and contribution to the development of the narrative framework of this review. Priority was given to key documents on diagnostic criteria for sarcopenia and frailty, such as EWGSOP2, AWGS2 and SDOC, as well as recent studies on muscle strength, lean body mass, weakness, frailty, mitochondrial biology and the epigenetics of exercise. Given the narrative and integrative nature of this review, no formal risk-of-bias assessment or quantitative synthesis was performed. However, to minimise selection and citation bias, studies were critically appraised based on methodological robustness, sample size, reproducibility of findings, and conceptual contribution to the proposed integrative framework. The synthesis process followed an inductive approach, whereby evidence was organised thematically and iteratively until conceptual saturation was achieved across the domains of physical performance, biological ageing and genetic–environmental interactions.

A narrative review methodology was deliberately chosen over a systematic or scoping review for three reasons. First, the aim of this work is conceptual integration rather than quantitative estimation of a specific effect size, which is the natural target of a systematic review. Second, the fields synthesised here (exercise genomics, geriatric physiology, gut-microbiota research, epigenetic ageing and evolutionary biology) use heterogeneous terminologies, outcomes and study designs that resist standardised pooling; a scoping review could map their extent, but would not achieve the mechanistic synthesis we pursue. Third, several of the key concepts discussed (functional reserve, pleiotropy, life-course trajectories) are inherently theoretical and integrative and are best treated through narrative appraisal of high-quality primary and secondary evidence. Nonetheless, to mitigate the limitations of narrative reviews (selection bias, absence of quality grading and lack of reproducibility) we structured the search into four conceptual blocks, prioritised high-impact primary studies and international consensus documents, appraised each cited study on methodological grounds, and made our inclusion criteria explicit. We acknowledge, however, that no formal risk-of-bias assessment was performed and that the strength of the individual claims made throughout this review should be interpreted accordingly.

The evidence identified was therefore organised around three main themes: shared functional phenotypes, converging biological pathways, and genetic–environmental modulators of functional reserve.

## 3. The Link Between Performance and GMDs: Common Phenotypes

In the field of sport, it is common to assess performance in terms of the primary metabolic and physical demands of the activity in question, such as cardiorespiratory endurance, maximum strength, muscular power, training adaptation or mechanical efficiency [15,27]. In people with geriatric motor dysfunctions (GMDs), physical abilities are usually assessed using specific tests, such as the Short Physical Performance Battery (SPPB), the Five Times Sit-to-Stand Test (5STS), measurement of walking speed, or handgrip strength [28]. Although the terminology used may differ, many of these tests or indicators are based on the same biological systems or global physical capability [29,30].

For example, cardiorespiratory endurance depends on the integration of oxygen transport and utilisation, ranging from cardiovascular function to the muscle’s oxidative capacity. In athletes, these capabilities are commonly expressed as maximum oxygen uptake (VO_2max_), running economy or time to exhaustion [15]. In an older person, the same biology determines the ability to walk, climb stairs or recover from an acute illness [28]. This reflects a systems-level continuity in aerobic capacity, where maximal physiological performance in youth transitions into submaximal functional independence in older age [14]. Similarly, muscle strength and power act as decisive factors in many sports, but also in the ability to stand up from a chair, prevent a fall or maintain a steady gait [31].

Importantly, endurance performance and strength/power performance should not be treated as biologically equivalent when considering their translation into ageing phenotypes. Skeletal muscle fibres exhibit asymmetric age-related vulnerability: fast-twitch (Type II) fibres, which underlie explosive strength and power output, undergo preferential atrophy, denervation and loss with ageing, whereas slow-twitch (Type I) fibres, more relevant to oxidative endurance, are comparatively preserved [17,20]. Consequently, the biological mechanisms supporting strength and power (including sarcomeric integrity, motor-unit remodelling, satellite cell reserve and mechanotransduction) overlap more directly with mechanisms involved in sarcopenia, mobility limitation and fall risk. By contrast, mitochondrial and oxidative pathways relevant to endurance performance are more closely related to cardiorespiratory resilience and systemic metabolic health. Recognising this dual axis clarifies why interventions targeting Type II fibres (resistance and power training) are particularly effective against sarcopenia and falls, whereas endurance-oriented interventions confer their strongest benefit on mortality and cardiometabolic ageing.

The trainability of physical capacities adds yet another layer of information to this continuum. For example, the HERITAGE study showed that the response of VO_2max_ to a standardised aerobic training programme exhibits considerable inter-individual variability, with a genetic component that could account for up to 49% of the variance in VO_2max_ trainability [32]. This inter-individual variability in training responsiveness supports the concept of “functional reserve plasticity”, whereby the adaptive capacity of physiological systems is partially genetically determined and expressed across both athletic and ageing populations [33,34]. In this regard, functional reserve plasticity may also be relevant in older adults: two individuals exposed to the same exercise stimulus, such as a strength training programme, may show different increases in muscle mass, strength or physical function. This variability is likely to reflect the combined influence of previous activity history, baseline functional status, metabolic health, comorbidities, nutrition and genetic predisposition.

When we consider the extreme end of physical capabilities in old age, one of the most defining characteristics is frailty. One possible criterion for identifying it is the Fried Phenotype, which groups together five key criteria: weight loss, exhaustion, weakness, slowness and a low level of physical activity; a person is considered frail if they meet three or more of these criteria [35]. Another widely used measure is the Rockwood Frailty Index, which focuses on the accumulation of various issues affecting an individual’s overall health and enables classification on a frailty scale [36]. These two operational frameworks capture complementary dimensions of frailty, with the Fried Phenotype emphasising physical frailty as a biological syndrome, and the Rockwood index conceptualising frailty as an accumulation of multidimensional deficits reflecting systemic decline [35,36]. Both methods reveal two sides of the same coin: on the one hand, measurable physical decline, and on the other, the loss of the ability to perform various everyday tasks or the development of dependency.

Another condition that has gained significant importance in recent years is sarcopenia, initially understood as the loss or lack of muscle mass in older people. According to the consensus established by the European Working Group on Sarcopenia in Older People (EWGSOP2), low muscle strength is the initial criterion, followed by confirmation of low muscle quantity or quality, and finally by an impact on physical performance that increases the severity of the diagnosis [37]. The AWGS2 Asian consensus takes the same approach, adapting the various diagnostic thresholds to Asian anthropometric characteristics using a community-based screening algorithm [38]. Furthermore, the SDOC (Sarcopenia Definition and Outcomes Consortium) proposes that handgrip strength and walking speed should be considered as primary diagnostic criteria for clinical trials, thereby prioritising muscle function over muscle mass [39]. All three consensus statements agree on the importance of strength and speed as key predictors, as these are essential abilities in a wide range of strength- and/or power-based sports. Collectively, these definitions reflect a conceptual shift from a morphology-centred view of sarcopenia towards a function-centred paradigm, aligning clinical diagnosis more closely with performance-based metrics used in sports science.

**Figure 2:**
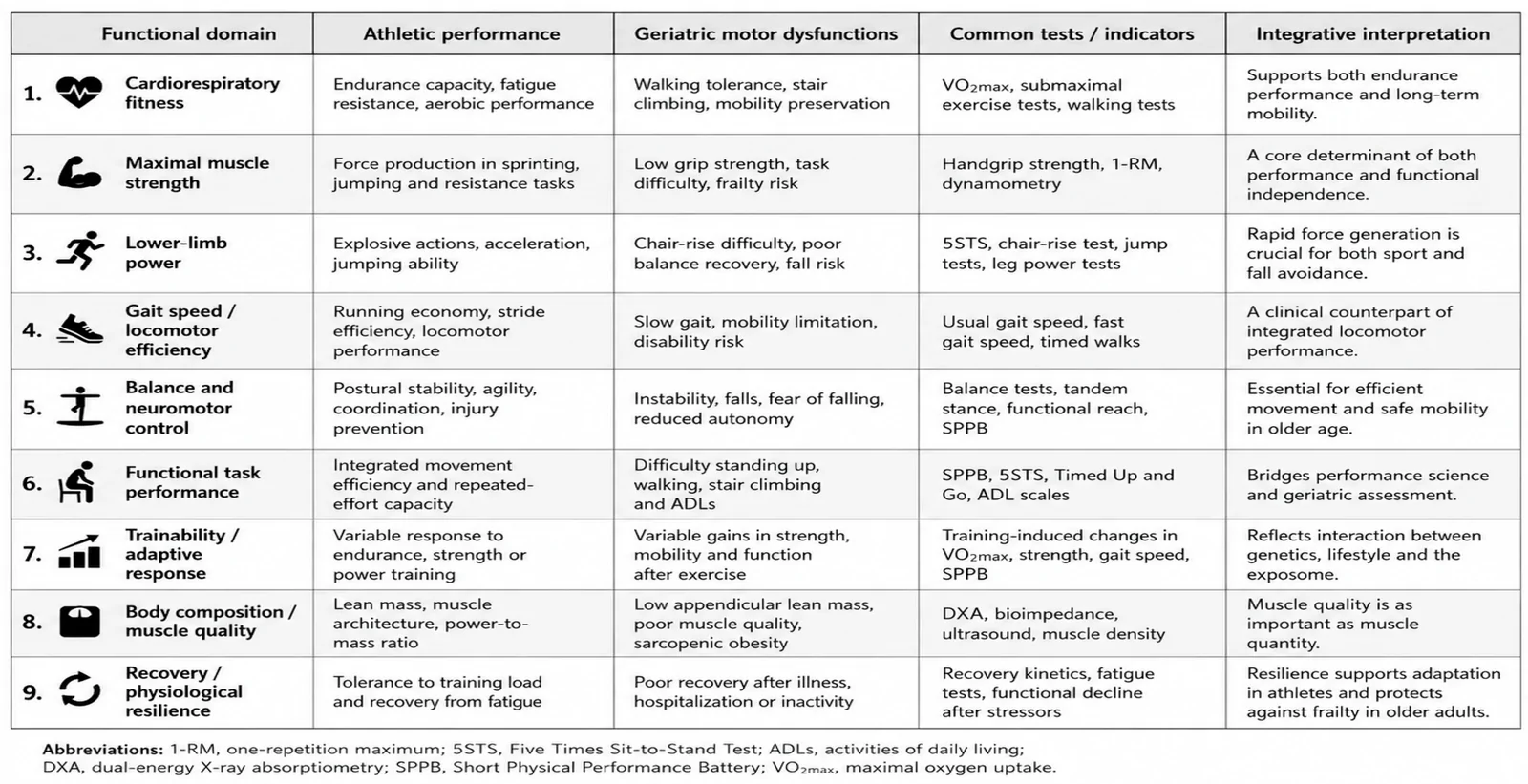
Functional phenotypes common to both athletic performance and geriatric motor dysfunctions. Summary of the main functional domains linking athletic performance to geriatric motor dysfunctions. Although sports science and geriatric assessment often use different terminology, both fields evaluate overlapping physiological systems, including cardiorespiratory fitness, muscle strength, lower-limb power, gait efficiency, balance, performance of functional tasks, trainability, muscle quality, and physiological resilience.

Finally, lower-limb weakness can be identified using measures such as gait speed, the SPPB or the 5STS and is associated with disability, institutionalisation and mortality. Taken together, the GMDs reflect a biological reality of the same process: functional and physical decline over the years (Figure 2) [40].

Conceptually, functional reserve corresponds to the difference between an individual’s maximal physiological capacity (peak VO_2max_, peak grip strength, peak lower-limb power) and the minimal capacity required to perform activities of daily living without disability. Operationally, this reserve can be estimated in three complementary ways. First, cross-sectional reserve can be approximated by comparing an individual’s current capacity with age- and sex-specific normative values. Second, longitudinal reserve can be evaluated by quantifying the rate of change in strength, gait speed or SPPB performance across repeated assessments. Third, resilience-based reserve can be estimated from the response to a standardised stressor, such as recovery after acute illness, surgery or a supervised exercise intervention, thereby reflecting the ability to buffer physiological perturbations. Emerging composite metrics, including intrinsic capacity as defined by the WHO, physical resilience scores and biological-age indicators derived from DNA methylation (see Section 5.7), offer complementary experimental proxies of functional reserve and are increasingly used in ageing research [6,7,41].

## 4. The Genetic Architecture Underlying Physical Performance and Decline: From Candidate Genes to Polygenic Traits

Research into the genetics of athletic performance gained prominence following the identification of early associations involving variants such as the insertion/deletion polymorphism in *ACE* and the R577X polymorphism in *ACTN3* [42,43]. *ACE* has been studied for its potential role in endurance or strength sports, with results varying over time; whereas *ACTN3* is particularly associated with performance in strength and/or power sports [44,45,46]. This latter association has been repeatedly re-evaluated in recent meta-analyses. The recent meta-analysis by Chelly and colleagues, incorporating case–control data published up to 2025, confirmed a modest but consistent over-representation of the RR/RX genotypes among elite power athletes across ethnicities, while acknowledging that the effect size at the individual level is insufficient for talent-identification purposes [47]. Another systematic review and meta-analysis of the *ACTN3* polymorphism frequency across Brazilian populations documented substantial inter-population heterogeneity that must be accounted for in comparative studies [48]. More broadly, meta-analyses of multiple candidate polymorphisms indicate that no single variant reliably discriminates athletes from non-athletes, reinforcing the transition from candidate-gene models to polygenic frameworks [49].

This same logic can be applied to GMDs: variants that may promote a greater response to hypertrophy, oxidative efficiency or response to training could enhance the ability to maintain strength or mobility in old age. However, candidate-gene studies have certain limitations: small sample sizes, small effects, replication issues and a tendency to overinterpret the role of isolated variants. These studies have been useful for identifying potential biological pathways involved, but despite this, they are not sufficient to explain the complexity of performance or its role in GMDs.

The emergence of GWASs enabled a shift from a candidate-gene approach to a polygenic approach, where specific traits can be studied in an agnostic manner with very broad genomic coverage. Traits related to muscle strength, lean body mass, walking speed, or response to exercise appear to be highly polygenic, depending on many variants with very small individual effects. For example, the GWAS conducted by Willems et al. in over 195,000 participants identified 16 significant loci associated with grip strength, providing the first evidence of Mendelian randomisation of a causal effect of muscle strength on fracture risk [25]. Tikkanen and colleagues, using data from UK Biobank, confirmed a genetic architecture distributed across loci involved in sarcomere organisation, calcium signalling and neurological development [50]. Jones and colleagues recently published one of the first genome-wide association studies (GWASs) specifically focusing on muscle weakness in older adults, involving around 256,000 participants. They identified 15 independent loci with genes implicated in neurological and muscular function and provided evidence, through Mendelian randomisation, of a causal link between weakness and frailty [26]. At the same time, other GWASs of appendicular lean body mass in the UK Biobank identified more than 250 associated SNPs, implicating muscular and neurological signalling pathways [51]. Finally, previous studies on body composition have identified the Wnt/β-catenin pathway and bone remodelling as key factors in determining lean body mass [52]. This growing body of evidence highlights the need to move beyond the concept of a ‘performance gene’ or a ‘sarcopenia gene’, and to focus instead on studying the networks underlying musculoskeletal development, neuromuscular signalling, metabolism and cellular regulation.

Importantly, many of the molecular pathways associated with elite physical performance overlap with the biological hallmarks of ageing. Mechanisms involving mitochondrial biogenesis, proteostasis, autophagy, cellular senescence, chronic low-grade inflammation and genomic stability are critically involved both in exercise adaptation and in age-related functional decline [53,54]. Exercise has been proposed as one of the most potent non-pharmacological modulators of these hallmarks, suggesting that some exercise-related genotypes may exert pleiotropic effects across the lifespan by simultaneously influencing athletic capacity and biological ageing trajectories.

Polygenic risk scores (PRSs) have emerged as an attempt to synthesise precisely this genetic complexity, integrating the effect of various variants into an estimate of predisposition to a particular trait, phenotype or condition. Theoretically, these scores could help identify individuals with lower muscle reserve or a higher risk of functional decline before any symptoms appear. In practice, their usefulness remains limited, as they can only account for a portion of the variance, have been developed primarily in European populations, and offer only modest improvement over simple functional markers such as grip strength or walking speed. For example, the LASA study found no association between the PRSs established for grip strength and frailty in the subjects, whereas direct measures of strength did show a robust association, suggesting that non-genetic factors accounted for much of the association [55]. Therefore, the current value of PRSs related to muscle function is greater as a research tool than as a population-based screening tool. Beyond grip strength, recent efforts have begun to build genome-wide polygenic scores that predict muscle strength, common disease risk and lifespan simultaneously. Herranen and colleagues developed a PRS for handgrip strength using the FinnGen cohort and demonstrated that low genetically predicted strength predicts risk for cardiovascular disease, type 2 diabetes and all-cause mortality across the life course, with evidence of significant sex-by-PRS interactions [56]. Multi-trait GWAS analyses of sarcopenia-related traits in UK Biobank have further expanded the catalogue of susceptibility loci [57]. However, several limitations constrain the current clinical translation of PRSs: (i) transferability across ancestries remains poor because most discovery cohorts are of European descent; (ii) absolute predictive gain over simple functional markers such as grip strength or gait speed remains modest, as confirmed by longitudinal cohorts such as LASA [55]; (iii) PRSs do not capture gene–environment interactions, which are particularly relevant for exercise responsiveness; and (iv) their communication to non-genetic professionals and to patients requires careful framing to avoid deterministic interpretations. Consequently, the near-term utility of muscular PRSs lies in identifying subgroups at elevated risk within research cohorts and in enriching clinical trial populations, rather than in replacing functional assessment.

Recent advances in genome-wide association studies and polygenic risk score methodologies further support the notion that physical performance and ageing phenotypes share a highly polygenic architecture. Rather than depending on single “performance genes”, complex traits such as muscle strength, aerobic fitness, frailty or walking speed are now understood to result from the cumulative interaction of thousands of variants with small individual effects [50,58]. This paradigm shift has substantial implications for exercise prescription and healthy ageing, opening the possibility of precision exercise medicine approaches tailored according to individual biological responsiveness and functional reserve profiles.

Nevertheless, genetic predisposition alone is insufficient to explain variability in performance or ageing outcomes. Environmental exposures across the life course, including nutrition, physical activity, sleep, psychosocial stress, socioeconomic circumstances and chemical or physical exposures, interact with biological systems and may modify gene expression through epigenetic, transcriptomic and metabolic mechanisms [59]. This interaction between genome and exposome may partially explain why individuals with similar genetic backgrounds can exhibit markedly different ageing trajectories or exercise responses. Accordingly, ageing-related motor dysfunctions should not be interpreted solely as consequences of chronological ageing, but rather as emergent phenotypes resulting from cumulative gene–environment interactions over time.

Emerging multi-omics approaches integrating genomics, epigenomics, metabolomics, proteomics and gut microbiome profiling are beginning to provide a more comprehensive understanding of the biological networks underlying both elite performance and functional decline [60,61]. These integrative models may help identify shared molecular signatures associated with adaptability, recovery capacity and neuromuscular resilience, thereby offering novel biomarkers for both sports performance optimisation and early detection of age-related functional deterioration.

Furthermore, there are also sex differences in relation to the development of GMDs. Skeletal muscle biology, athletic performance and the epidemiology of GMDs are all sexually dimorphic. Women exhibit lower absolute muscle mass and strength than men across the life course, but a more favourable metabolic and oxidative profile, and a higher relative preservation of Type I fibres [62]. Sarcopenia and frailty show sex-specific prevalence patterns: after adjusting for absolute cut-points, older women present with a higher prevalence of frailty, whereas sarcopenia diagnostic rates depend heavily on the reference thresholds applied [63,64]. At the genetic level, sex-stratified analyses remain relatively rare. Herranen and colleagues reported that a polygenic score for handgrip strength explained 5.2% of the phenotypic variance in women versus 4.3% in men, with a significant interaction between sex, PRS and leisure-time physical activity [65]. Sex-specific effects of PRSs on metabolic outcomes have also been reported in the FinnGen cohort [56]. Gim and colleagues, using the Korean Genome and Epidemiology Study, provided one of the few large GWASs with sex-stratified models for lean muscle mass, identifying partially non-overlapping loci in men and women [66]. Beyond the nuclear genome, sex hormones (oestrogens, androgens) exert powerful modulatory effects on muscle protein synthesis, satellite cell dynamics and neuromuscular junction integrity; menopause-associated declines in oestrogen have been associated with accelerated losses of both muscle mass and function [62,67].

## 5. Key Biological Pathways: The Mechanisms Behind the Overlap

Although the pathways discussed below are presented separately for explanatory purposes, they operate as highly interconnected biological networks rather than independent mechanisms. Recent advances in systems biology suggest that functional reserve emerges from the collective behaviour of multiple interacting systems, including mitochondrial energetics, neuromuscular integrity, immune regulation, mechanotransduction and tissue repair. Consequently, athletic performance and geriatric motor dysfunctions may not represent distinct biological entities but rather different phenotypic expressions of the same underlying network architecture operating under different levels of physiological resilience [14,68]. Athletic performance and GMDs may therefore represent different phenotypic expressions of partially shared network architecture operating under different levels of physiological resilience.

Many of the biological pathways studied in sports science are also linked to age-related functional decline [53,69]. The relationship cannot therefore be reduced to a linear correspondence between isolated “performance genes” and “sarcopenia genes”. Classic candidate genes, such as *ACTN3*, *ACE* and *PPARGC1A*, should be interpreted alongside the loci and genes identified through genome-wide association studies and more recent functional research, including *IRS1*, *PIK3R1*, *FTO*, *STC2*, *CPNE1*, *NEB*, *RIF1*, *HLA-DRB1*/*DRB5*, *IL6R*, *FOXP1*, *ZBTB38*, *PIEZO1*, and *YAP1*/*WWTR1* or components of the PINK1/Parkin pathway; this would help to explain why phenotypes such as strength, power, lean mass, walking speed and frailty may share a distributed and highly interconnected biological basis [51,70,71].

Among the relatively well-established mechanisms are PGC-1α-mediated mitochondrial biogenesis, anabolic signalling via the IGF-1/PI3K/Akt/mTOR axis, protein degradation via the ubiquitin–proteasome system, low-grade chronic inflammation, and age-related deterioration in satellite cell function. These mechanisms are supported, to a greater or lesser extent, by converging evidence from experimental models, observational studies in humans, genetic analyses and interventions relating to exercise or nutrition. Conversely, emerging areas include the systemic actions of mitochondria-derived peptides, such as MOTS-c; epitranscriptomic regulation via the m^6^A-associated mechanism; the contribution of mechanosensitive channels, such as PIEZO1; the redox regulation of cellular senescence and the senescence-associated secretory phenotype; metabolites derived from the gut microbiota, such as urolithin A and tryptophan derivatives; and biomarkers of biological ageing based on DNA methylation.

### 5.1. Mitochondrial Function, Redox Homeostasis and Mitophagy

In athletes, the oxidative capacity of the muscle determines endurance, recovery and tolerance to repeated exertion. In older adults, mitochondrial dysfunction is associated with reduced ATP production, increased oxidative stress, impaired mitophagy, greater accumulation of mutated mtDNA and a decline in muscle quality [72]. PGC-1α acts as a key regulator of mitochondrial biogenesis and links aerobic exercise, oxidative metabolism and muscle ageing [69]. Exercise activates, in a coordinated manner, mitochondrial biogenesis via PGC-1α and selective mitophagy via PINK1/Parkin, ensuring the renewal of a functional mitochondrial network [73].

At the genetic level, this pathway can be organised into three modules. The first relates to mitochondrial biogenesis, regulated by *PPARGC1A*/PGC-1α, *NRF1*, *NRF2*/*GABPA*, *TFAM*, *ESRRA* and *SIRT1*/AMPK, which control the expansion of the mitochondrial network, mitochondrial genome transcription and oxidative capacity. Importantly, NRF2/*GABPA*, involved in mitochondrial biogenesis, is mechanistically distinct from *NFE2L2*/Nrf2, the KEAP1-regulated transcription factor that coordinates antioxidant and cytoprotective responses. Accordingly, NRF2/*GABPA* is considered here in the context of mitochondrial biogenesis, whereas *NFE2L2*/Nrf2 is discussed in relation to redox homeostasis and antioxidant defence. The second corresponds to mitochondrial dynamics, dependent on fusion and fission genes such as *MFN1*, *MFN2*, *OPA1*, *DNM1L*/DRP1 and *FIS1*, whose balance maintains a mitochondrial network that is adaptable to training and resistant to metabolic stress. The third module relates to mitophagy and mitochondrial quality control, where *PINK1*, *PRKN*/Parkin, *BNIP3*, *BNIP3L*/NIX, *FUNDC1*, *SQSTM1*/p62, *OPTN* and LC3/*MAP1LC3B* enable the recognition and elimination of damaged mitochondria [71,74]. Mitochondrial biogenesis promotes endurance, recovery and energy efficiency in early life, whilst the loss of mitophagy, mitochondrial fragmentation and the accumulation of damaged mtDNA contribute to fatigue, reduced anabolic capacity, sterile inflammation and a decline in muscle quality in old age. Furthermore, the PGC-1α–AMPK–SIRT1–PINK1/Parkin pathway directly links exercise, energy restriction, selective autophagy and muscular ageing. Therefore, mitochondrial function should not be viewed solely as a pathway for aerobic performance, but as a cellular maintenance system that determines functional reserve throughout the entire life course.

Other key genes involved in these processes include those of the PPAR (peroxisome proliferator-activated receptor) family, such as *PPARA*, *PPARD* and *PPARG*, and the aforementioned *PPARGC1A*, which encodes PGC-1α. The peroxisome proliferator-activated receptor alpha is expressed in skeletal muscle and brown adipose tissue, amongst other tissues, playing an important role in fatty acid oxidation and glucose metabolism. Under conditions of energy deprivation, the *PPARA* gene is activated to promote the uptake and utilisation of fatty acids as an energy source. *PPARD* is probably the subtype with the greatest direct relevance in skeletal muscle, where it has been linked to the regulation of lipid oxidation, mitochondrial biogenesis, resistance to fatigue and the transition towards a more oxidative phenotype of muscle fibres. *PPARG* is most highly expressed in adipose tissue and is a key regulator of adipogenesis, lipid storage and insulin sensitivity. Although its direct role in muscle fibre is less dominant than that of *PPARA* or *PPARD*, it indirectly influences muscle function by modulating lipid distribution, systemic inflammation, adipokine secretion, and the risk of lipotoxicity and insulin resistance. Finally, PGC-1α acts as an integrator of all these responses. It functions as a co-activator of multiple transcriptional programmes related to mitochondrial biogenesis, oxidative respiration, angiogenesis, defence against oxidative stress, and the conversion to fibres that are more resistant to fatigue. Exercise, particularly aerobic training and repeated contractile stimuli, induces the expression of PGC-1α in skeletal muscle, facilitating the expansion of the mitochondrial network and improving oxidative capacity [75]. Taking all of the above into account, greater efficiency in substrate utilisation, improved mitochondrial plasticity and a more robust adaptive response to training could contribute both to performance and to reduced vulnerability to the onset of age-related motor dysfunctions.

Beyond energy production, mitochondria function as signalling organelles through mitochondria-derived peptides, including humanin, MOTS-c and small humanin-like peptides. These molecules have been implicated in insulin sensitivity, inflammatory regulation, metabolic stress responses and exercise-related adaptation. MOTS-c, in particular, represents a potential link between mitochondrial communication, metabolic resilience and functional ageing, although its clinical and predictive relevance in humans remains to be established [76,77,78].

A further dimension of this mitochondrial pathway is redox signalling and its relationship with the endogenous antioxidant response. Mitochondrial reactive oxygen species (ROS) are not merely toxic by-products; at low or moderate levels, they act as second messengers that activate adaptive programmes, including mitochondrial biogenesis and stress resistance [79,80,81]. However, the age-related imbalance between ROS production and antioxidant neutralisation leads to a state of chronic oxidative stress that progressively compromises mitochondrial and cellular function. The Nrf2/KEAP1 pathway is fundamental to the endogenous antioxidant response: under conditions of oxidative stress, Nrf2 translocates to the nucleus and induces the transcription of cytoprotective genes encoding superoxide dismutase, catalase, glutathione peroxidase and heme oxygenase-1 [82,83]. With ageing, both the basal activity of Nrf2 and its inducibility decline, reducing the capacity to buffer mitochondrial ROS and contributing to the accumulation of oxidative damage in skeletal muscle [84,85]. It is crucial to note that mitochondrial ROS also act as upstream activators of the NLRP3 inflammasome, providing a direct mechanistic link between mitochondrial dysfunction, oxidative stress and the chronic inflammatory state characteristic of inflammageing [86,87]. Exercise, by transiently elevating ROS and activating Nrf2 in a hormetic manner, represents a major non-pharmacological strategy for supporting redox homeostasis and mitigating this mitochondria-to-inflammation cascade across the life course [88,89,90].

### 5.2. Anabolic–Catabolic Balance

Muscle mass and strength depend on the balance between protein synthesis and breakdown, which is regulated by the IGF-1/PI3K/Akt/mTOR, myostatin/activin, ubiquitin–proteasome (FoxO-regulated atrogenes) and autophagy pathways [91]. In early life, efficient anabolic signalling facilitates hypertrophy and adaptation to training. In old age, anabolic resistance due to reduced activation of mTORC1 and myofibrillar protein synthesis in response to equivalent stimuli, reduced mechanical stimulation and the activation of catabolic pathways (atrogin-1, MuRF-1) contribute to the loss of muscle strength and mass.

At the genetic level, this pathway includes a central anabolic axis comprising *IGF1*, *IGF1R*, *INSR*, *IRS1*, *PIK3R1*, *AKT1*, *MTOR*, *RPTOR*, *RHEB*, *EIF4EBP1* and *RPS6KB1*, responsible for activating protein translation and myofibrillar hypertrophy. Conversely, the catabolic axis includes *FOXO1*, *FOXO3*, *FBXO32*/atrogin-1, *TRIM63*/MuRF1, *MSTN*, *ACVR2A*/*ACVR2B*, *SMAD2/3*, *GDF15* and autophagy genes such as *BECN1*, *ATG5*, *ATG7* and *ULK1*. Recent genetic evidence identifies *IRS1* and *PIK3R1* as particularly relevant genes, as their variants are associated with lean mass, fat mass and body composition, supporting a muscle–adipose pleiotropy linking sarcopenia, sarcopenic obesity and insulin resistance [70,92,93]. This metabolic link is reinforced by Mendelian-randomisation studies, which suggest a bidirectional relationship between traits associated with sarcopenia and type 2 diabetes, supporting the view that insulin resistance is not merely a comorbidity, but a potential causal modulator of muscle function decline [94].

Therefore, strength performance and the prevention of age-related muscle loss depend not only on muscle mass, but also on the sensitivity of muscle tissue to mechanical, nutritional and hormonal anabolic signals. Anabolic resistance in old age can be understood as a loss of efficiency in the insulin/IGF-1–PI3K–Akt–mTOR axis, exacerbated by inflammation, inactivity, gut dysbiosis and reduced availability of essential amino acids. Conversely, strength and power training act as an intervention capable of reactivating this axis, partially inhibiting FOXO/atrogenic factors and restoring myofibrillar protein synthesis, even in older age.

Recent geroscience frameworks propose that alterations in proteostasis represent a central hallmark linking anabolic resistance and age-related functional decline. Beyond reduced protein synthesis, ageing muscle exhibits impairments in protein folding, chaperone activity, autophagic flux and proteasomal degradation efficiency, leading to the accumulation of dysfunctional proteins [69]. Exercise appears capable of partially restoring proteostatic mechanisms through coordinated activation of autophagy, heat-shock proteins and lysosomal pathways, suggesting that muscle quality may depend as much on protein turnover efficiency as on net muscle mass accretion [95].

### 5.3. Inflammageing and Immunometabolism

Intense exercise triggers transient inflammatory responses that form part of the adaptation process; ageing, on the other hand, is accompanied by a persistent state of low-grade inflammatory activation known as inflammageing [24,96]. When pro-inflammatory cytokines such as IL-6 or TNF-α remain chronically elevated, they interfere with anabolic signalling, promote catabolism and contribute to functional decline. This link between ageing, cardiovascular disease, frailty and inflammation positions GMDs as a multisystemic syndrome, not just a muscular one [24].

GWASs on frailty and muscle weakness have identified the involvement of regions within the major histocompatibility complex, such as *HLA-DRB1*, *HLA-DRB5*, *HLA-DQB1*, *BTNL2* and *TNXB*, as well as inflammatory signalling genes such as *IL6*, *IL6R*, *IL10*, *TNF*, *TNFSF9*, *NFKB1*, *RELA*, *TLR4*, *NLRP3*, *JAK2* and *STAT3*. The *IL6R* rs2228145 variant, for example, has been used in Mendelian-randomisation studies to explore the causal contribution of IL-6 signalling to inflammatory diseases and age-related traits. At the level of muscle function, these pathways link antigen presentation, immunosenescence, systemic inflammation, anabolic resistance and neuromuscular deterioration [26,97,98].

A very illustrative example is that of IL-6: it is important to distinguish between IL-6 as an acute exercise-induced myokine, with context-dependent metabolic and anti-inflammatory effects, and chronically elevated IL-6 associated with inflammageing, which is linked to catabolism, frailty and poorer physical performance. This duality is important in our context: the same molecule can contribute to beneficial adaptation or functional decline depending on the temporal pattern, tissue source and metabolic context. Thus, the overlap between performance and GMDs is not explained solely by muscle genes, but also by immunometabolic genes that regulate the body’s ability to resolve inflammation following mechanical stress and prevent repair from becoming chronic damage.

The relationship between inflammation and functional ageing extends beyond cytokine concentrations. Ageing is accompanied by profound remodelling of the immune system, including thymic involution, T-cell exhaustion, altered macrophage polarisation and expansion of senescent immune cell populations [99]. This phenomenon, termed immunosenescence, contributes to impaired tissue repair, chronic inflammation and reduced responsiveness to exercise interventions. Interestingly, lifelong physical activity appears to attenuate several features of immunosenescence, suggesting that exercise may preserve immune adaptability in a manner analogous to its effects on skeletal muscle and cardiovascular function [100].

This immune remodelling is closely coupled to redox imbalance. ROS generated by dysfunctional mitochondria and activated immune cells can oxidise DNA, lipids and proteins, generating damage-associated molecular patterns (DAMPs) that activate pattern-recognition receptors and downstream inflammatory cascades, including NF-κB and the NLRP3 inflammasome [87,101,102]. Activated NF-κB promotes the transcription of pro-inflammatory cytokines (IL-6, TNF-α, IL-1β) whilst suppressing antioxidant gene programmes, perpetuating a redox-inflammatory loop that self-amplifies and intensifies with age. This cycle, often conceptualised as “oxi-inflamm-ageing”, is further sustained by declining endogenous antioxidant capacity and by age-related attenuation of the Nrf2/KEAP1 pathway, which normally coordinates the transcriptional induction of cytoprotective and antioxidant genes [82,84]. In the context of muscle ageing, oxidative stress may impair insulin/IGF-1–PI3K–Akt signalling through oxidative modifications of proteins and lipids, including protein carbonylation and lipid peroxidation, thereby contributing to anabolic resistance in parallel with cytokine-driven catabolic signalling [103].

Oxidative stress is also an important trigger of cellular senescence. Accumulated oxidative damage to DNA activates the p53/p21 and p16INK4a tumour suppressor pathways, leading cells into irreversible senescence and triggering the secretion of the senescence-associated secretory phenotype (SASP), which amplifies systemic inflammation and compromises the regenerative microenvironment available to satellite cells [104]. At the biomarker level, circulating markers of oxidative damage, including F2-isoprostanes, malondialdehyde (MDA) and 8-hydroxy-2′-deoxyguanosine (8-OHdG), have been reported alongside elevated levels of IL-6 and C-reactive protein in older adults with frailty or sarcopenia, reinforcing the notion that redox dysregulation and inflammatory activation are concurrent, mutually reinforcing processes, rather than independent phenomena [105,106].

### 5.4. Neuromuscular Integrity and Satellite Cell Reserve

Strength does not depend solely on muscle size: it requires motor neurons, a functional neuromuscular junction (NMJ), efficient motor-unit recruitment and motor control. The neuromuscular junction is one of the most vulnerable aspects of musculoskeletal ageing: partial denervation, motor endplate fragmentation and the loss of motor units explain why strength declines more rapidly than muscle mass [107]. At the same time, satellite cells lose their regenerative capacity with age. Sousa-Victor and colleagues demonstrated in aged mice that geriatric satellite cells can transition from reversible quiescence to a senescent state mediated by p16INK4a, thereby compromising muscle regeneration [108]. In athletic settings, differences in neuromuscular coordination and regenerative capacity may contribute to performance variability; in older age, deterioration of the same systems may increase weakness, mobility limitation and fall risk.

The stability of the motor endplate depends on the AGRN–LRP4–MUSK–DOK7 axis, together with RAPSN and subunits of the nicotinic acetylcholine receptor such as CHRNA1, CHRNB1, CHRND and CHRNE. These molecules regulate acetylcholine receptor clustering, postsynaptic maturation and the stability of the neuromuscular synapse. Ageing, by altering this architecture, reduces the reliability of neuromuscular transmission and contributes to denervation, the loss of type II fibres and a decline in muscle strength [109]. At the same time, the muscle’s regenerative reserve depends on genes such as *PAX7*, *MYOD1*, *MYF5*, *MYOG*, *MEF2C*, *NOTCH1*, *DLL1*, *WNT7A*, *TGFB1* and *CDKN2A*/p16INK4a. In early stages, efficient activation of satellite cells enables hypertrophy, repair following injury and adaptation to training; in old age, the senescence of these cells, reduced Notch signalling, increased TGF-β and the inflammatory environment reduce the capacity for regeneration. All this suggests that geriatric motor dysfunctions (GMDs) do not result solely from the loss of muscle tissue, but from the progressive degradation of the entire neuromuscular axis.

Emerging evidence suggests that neuromuscular ageing should not be viewed exclusively as a peripheral phenomenon. Structural and functional alterations within cortical motor networks, corticospinal pathways and sensorimotor integration circuits also contribute to age-related declines in strength, coordination and mobility [110]. Consequently, preservation of motor function across the lifespan may depend not only on muscle regenerative capacity but also on lifelong maintenance of neuroplasticity and central motor control [111,112].

### 5.5. Mechanotransduction

Muscle is a tissue that is sensitive to mechanical stress: it requires tension, stretching and contraction to maintain its structure. Mechanosensitive channels such as PIEZO1 are involved in translating physical forces into cellular signals; during myogenesis, PIEZO1 regulates the fusion and elongation of myotubes via Ca^2+^ flux and the RhoA/ROCK pathway [113]. Disuse of muscle tissue leads to atrophy, whereas strength training remains effective even in old age: mechanical loading is not merely an external intervention, but an essential molecular signal for maintaining function.

The mechanotransduction pathway can be divided into three genetic levels. The first corresponds to direct mechanical sensors, such as *PIEZO1*, *PIEZO2*, *ITGA7*/*ITGB1* integrins, *DMD* dystrophin, costamers and sarcomeric proteins such as *ACTN3*, *TTN* and *NEB*. The second corresponds to intracellular transducers such as *PTK2*/FAK, *RHOA*, *ROCK1/2*, MAPK, mTORC1 and the Hippo effectors *YAP1* and *WWTR1*/TAZ. The third corresponds to final adaptive responses: protein synthesis, extracellular matrix remodelling, myogenic differentiation and local angiogenesis [114].

Mechanical sensitivity determines the extent to which a muscle ‘translates’ a given external load into growth, repair or maintenance. A young muscle subjected to training activates mTOR, YAP/TAZ and hypertrophy programmes; whereas an aged, inflamed or anabolism-resistant muscle may receive the same stimulus but with a diminished response. On the other hand, *NEB* encodes nebulin, a structural protein that regulates the length of thin filaments and sarcomeric stability; *RIF1* is involved in repair and genomic stability. Its association with muscle mass suggests that the mechanical quality of the muscle depends both on sarcomeric integrity and on the ability to maintain the cellular genome under repeated stress. Thus, mechanotransduction integrates not only the response to load, but also the structural capacity of the muscle to tolerate it without deteriorating.

Recent studies indicate that the extracellular matrix (ECM) should be considered an active participant in mechanotransduction rather than a passive structural scaffold [115]. Ageing-associated fibrosis, altered collagen cross-linking and changes in ECM stiffness modify force transmission and mechanosensory signalling, potentially reducing the effectiveness of anabolic stimuli [116]. Therefore, age-related impairments in muscle adaptation may arise not only from intracellular resistance to mechanical loading but also from alterations in the extracellular mechanical environment itself.

### 5.6. Myokines, Exerkines and Muscle–Organ Communication

Skeletal muscle is not only an effector organ but also an endocrine organ. The review by Severinsen and Pedersen lists more than 650 factors released by muscle during exercise, including IL-6 (which paradoxically has acute anti-inflammatory effects on myocytes), irisin, BDNF, myonectin, decorin and SPARC, with endocrine and paracrine actions on the liver, bone, adipose tissue and the brain [117]. This broadens the link between physical activity and health to a systemic level, integrating muscle into communication networks between organs, the disruption of which with age contributes to multisystemic frailty.

The myokine network can be viewed as a muscle–organ communication system that translates muscle contraction into systemic signals. Among the most relevant genes/factors are *IL6*, *FNDC5*/irisin, *BDNF*, *APLN*/apelin, *LIF*, *METRNL*, *SPARC*, *DCN*/decorin, *FST*/follistatin, *MSTN*/myostatin, *GDF15*, *ANGPTL4* and CTRP15/myonectin. In the context of performance, these signals promote metabolic adaptation, angiogenesis, substrate oxidation, neural plasticity and bone remodelling. In ageing, the loss of contractile mass, inactivity and inflammageing reduce the quality of the muscle secretome and disrupt communication with the liver, bone, adipose tissue, immune system and brain [118,119,120].

Sarcopenia, therefore, is not merely the loss of muscle as a mechanical tissue, but the loss of an endocrine organ. The decline in beneficial myokines and the increase in catabolic signals such as myostatin or GDF15 may contribute to multisystemic frailty, age-related anorexia, metabolic decline, reduced neuroplasticity and a poorer response to exercise. Conversely, regular training not only improves strength or VO_2max_, but also partially restores a more favourable secretory profile, transforming active muscle into a systemic modulator of health.

The concept of myokines has recently evolved into the broader framework of exerkines, encompassing bioactive molecules released during physical activity from multiple tissues including skeletal muscle, adipose tissue, liver, bone and the vascular endothelium [121]. This integrated signalling network mediates many of the systemic benefits of exercise and may explain why physical activity influences cognitive function, immune competence, metabolic health and longevity beyond its effects on muscle tissue alone [122,123].

Beyond their role as intercellular messengers, myokines and other exerkines may influence systemic redox homeostasis by transmitting the effects of muscle contraction to distal tissues [117,122]. Exercise-induced muscle contractions generate a transient and controlled increase in reactive oxygen species (ROS), which, through hormetic mechanisms, can activate the Nrf2/KEAP1 antioxidant pathway and PGC-1α-mediated adaptive programmes, thereby reinforcing endogenous antioxidant defences in skeletal muscle and, potentially, in distal tissues [79,124]. This exercise-driven antioxidant response contrasts with the less favourable secretory profile of inactive or ageing muscle, which may be characterised by increased catabolic or stress-associated factors such as myostatin and GDF15, together with altered expression of exercise-responsive myokines such as irisin and apelin [125,126]. Thus, the decline in muscle mass and contractile activity with ageing may progressively attenuate exercise-induced antioxidant signalling, contributing to systemic redox dysregulation and inflammageing. Regular strength and endurance training should therefore not be viewed solely as a strategy for preserving muscle function and the anabolic secretome, but also as a means of supporting systemic redox homeostasis and mitigating components of oxi-inflamm-ageing across the life course [89]. The molecular mechanisms linking ROS generation, Nrf2/KEAP1 signalling, NF-κB activation and the NLRP3 inflammasome are discussed in detail in Section 5.1 and Section 5.3.

### 5.7. Epigenetic Memory of Exercise

Exercise leaves stable epigenetic marks in skeletal muscle. Lindholm and colleagues described a coordinated reprogramming of the muscle methylome and transcriptome following aerobic exercise in humans, with changes at loci regulating oxidative metabolism [127]. Seaborne and colleagues demonstrated that muscle possesses an epigenetic memory of hypertrophy: previous episodes of training lead to persistent hypomethylation of anabolic genes, which facilitates muscle ‘recovery’ following periods of inactivity [128]. This capability has direct implications for veteran athletes, for rehabilitation following periods of immobilisation, and, conceptually, for the functional reserve model: early physical activity may leave molecular imprints that facilitate an adaptive response decades later.

In the study by Seaborne et al., genes such as *UBR5*, *RPL35A*, *SETD3*, *HEG1* and *PLA2G16* showed hypomethylation and increased expression following loading, with amplified responses during reloading; other genes such as *AXIN1*, *GRIK2*, *CAMK4* and *TRAF1* retained hypomethylated signatures during the unloading period, suggesting a molecular memory of the previous stimulus. Therefore, it is not merely a matter of ‘having trained before’, but of maintaining a muscle that is molecularly primed to respond better to new loads [128,129].

In addition to DNA methylation, epigenetic regulation of muscle involves histone remodelling, miRNAs and epitranscriptomic regulation via m^6^A. In this latter context, *FTO* emerges as a gene of particular interest for this manuscript, as it integrates adiposity, energy metabolism, muscle differentiation, mTOR–PGC-1α signalling and the cellular stress response. *FTO*, therefore, should not be confined to its classical role in obesity, but rather interpreted as an epitranscriptomic modulator of muscle homeostasis and a potential bridge between metabolism, hypoxia, myocellular senescence and GMDs.

From a conceptual standpoint, epigenetic memory provides molecular support for the life-course model: exercise undertaken in youth or adulthood may increase peak functional reserve, but may also leave adaptive marks that facilitate recovery following periods of inactivity in later life. This hypothesis does not imply determinism nor does it guarantee protection against the onset of any GMD, but it does reinforce the idea that the history of physical activity is inscribed in partially persistent molecular layers.

**Figure 3:**
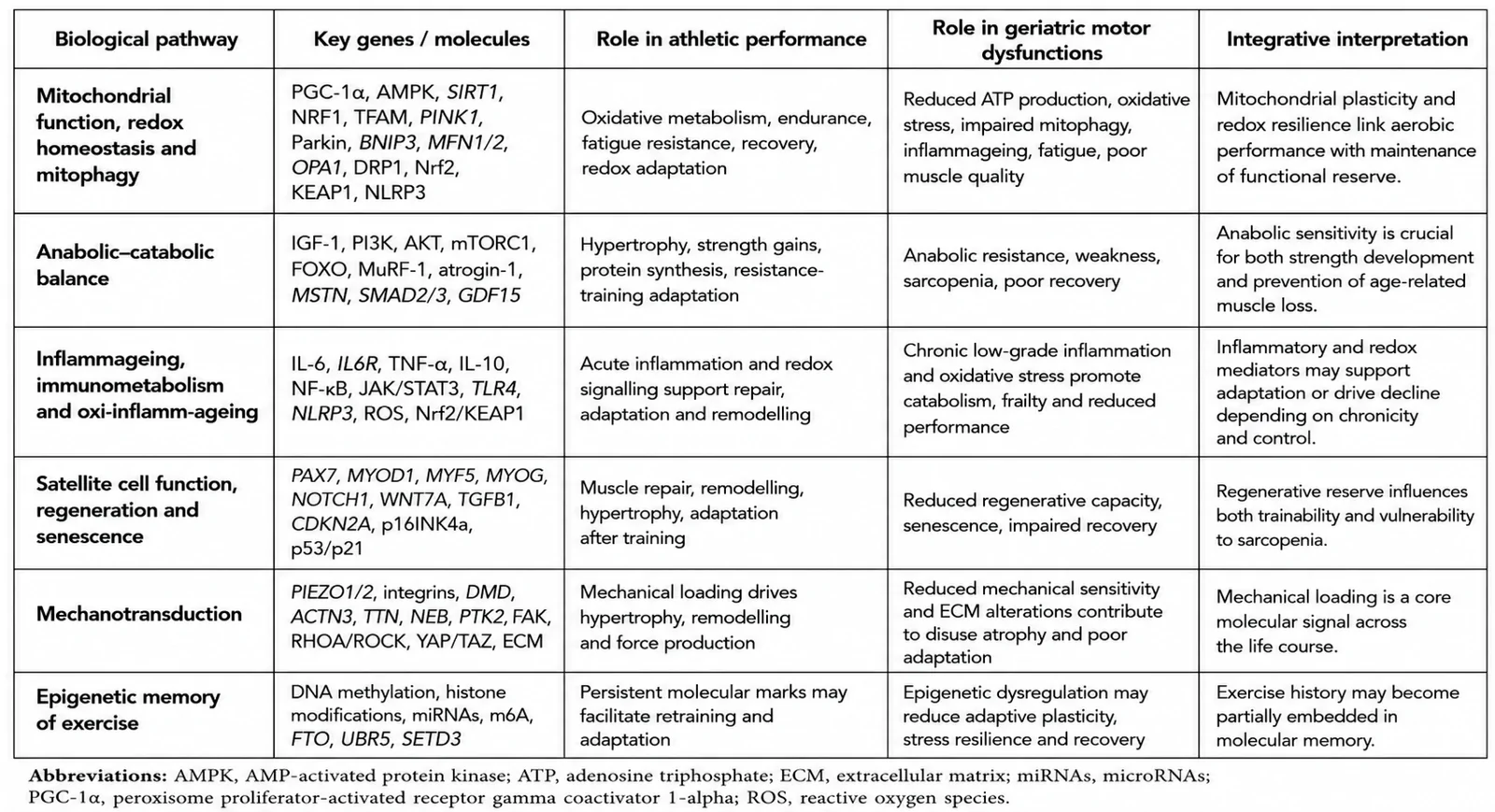
Shared molecular and cellular pathways linking athletic performance and geriatric motor dysfunctions. Summary of the main molecular and cellular pathways proposed to underlie the overlap between athletic performance phenotypes and geriatric motor dysfunctions across the life course. The table includes representative genes and molecules, their role in athletic performance, their role in geriatric motor dysfunctions, and an integrative interpretation for each pathway. The pathways shown comprise mitochondrial function, redox homeostasis and mitophagy; anabolic–catabolic balance; inflammageing, immunometabolism and oxi-inflamm-ageing; satellite cell function, regeneration and senescence; mechanotransduction; and epigenetic memory of exercise.

**Figure 4:**
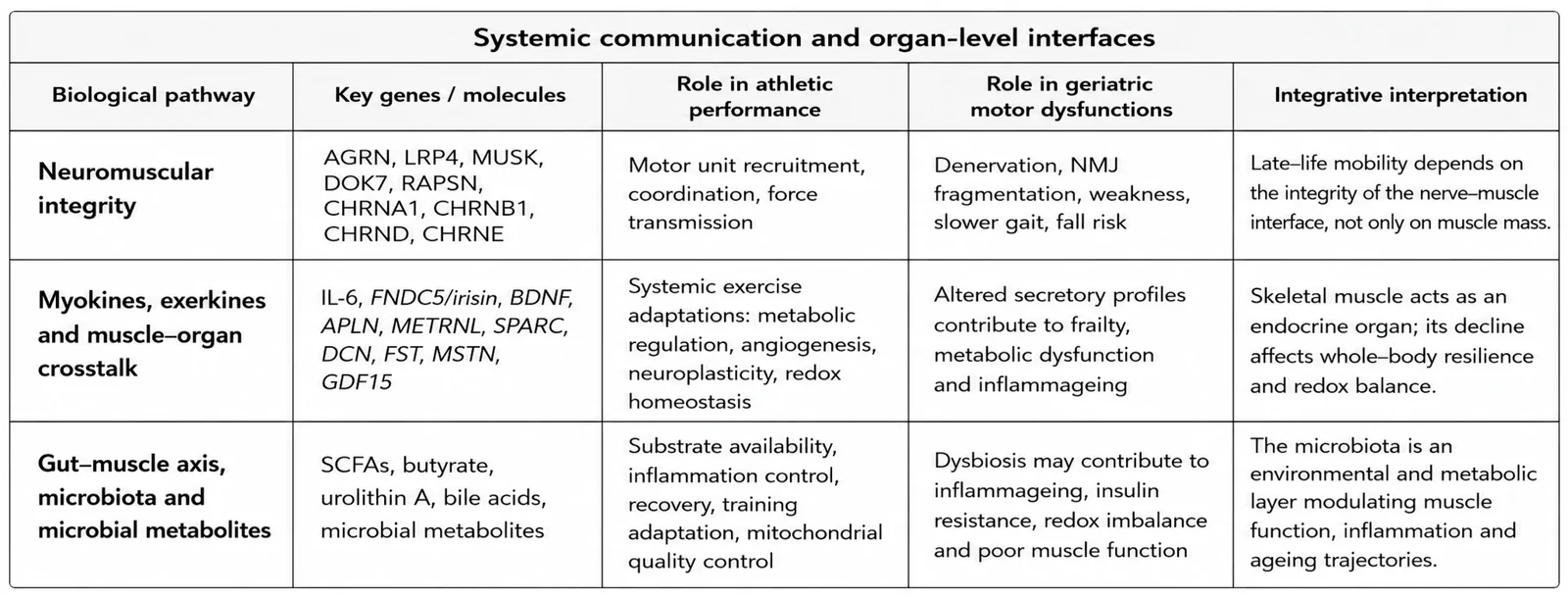
Shared systemic and organ-level pathways linking athletic performance and geriatric motor dysfunctions. Summary of the principal systemic and organ-level pathways linking athletic performance with geriatric motor dysfunctions. The table presents representative genes and molecules, their role in athletic performance, their role in geriatric motor dysfunctions, and an integrative interpretation for each pathway. The pathways shown include neuromuscular integrity; myokines, exerkines and muscle–organ crosstalk; and the gut–muscle axis, microbiota and microbial metabolites. Together with Figure 3, this figure illustrates the multilevel biological architecture shared by exercise adaptation, functional reserve and age-related motor decline.

The biological pathways mentioned above are summarised in Figure 3 and Figure 4.

Recent developments in biological-age estimation have strengthened the link between exercise, epigenetic regulation and GMDs. DNA methylation-based epigenetic clocks have evolved through three generations. First-generation clocks were trained to estimate chronological age from CpG methylation patterns and achieve high accuracy but capture only limited health-related information [130,131]. Second-generation clocks, PhenoAge and GrimAge, incorporate clinical or plasma-protein biomarkers alongside methylation and outperform first-generation models in predicting mortality, incident disease, cognitive decline, disability and, furthermore, frailty and physical function [132,133,134,135]. Third-generation biomarkers, epitomised by DunedinPACE, estimate the pace of biological ageing rather than cumulative epigenetic age, and have shown strong associations with morbidity, disability and mortality across independent European cohorts [136]. Emerging simplified and tissue-accessible methylation models may reduce the technical and economic barriers associated with biological-age assessment, although their capacity to predict clinically meaningful ageing outcomes remains to be established [137].

The relationship between epigenetic ageing and GMDs is supported by recent studies. Mak and colleagues, in the SATSA cohort and other European cohorts, demonstrated that DunedinPACE allows for tracking the temporal dynamics of frailty from middle age through old age, observing that a faster rate of epigenetic ageing precedes an increase in the Rockwood frailty index [138]. Tay and colleagues, in nonagenarians from the SG90 cohort in Singapore, demonstrated that grip strength, walking speed, and SPPB scores are inversely associated with GrimAge and DunedinPACE, providing evidence that DNA methylation clocks integrate the biology of physical function in the very elderly [139]. Beyond the specific contexts of each disease, Loh and colleagues reported that exercise interventions in older adults with myeloid neoplasms produced parallel reductions in GrimAge, PhenoAge, and DunedinPACE that correlated with simultaneous increases in grip strength, directly linking exercise-induced epigenetic rejuvenation to functional performance [140]. Cross-sectional population data from the Rhineland Study further show that physical activity measured with accelerometers is inversely associated with the acceleration of epigenetic ageing, consistent with a dose-dependent effect of daily activity on biological ageing [141].

Whether structured lifestyle interventions can slow or reverse epigenetic age is an active area of investigation. A recent randomised controlled trial by Nishimura and colleagues in men aged ≥50 years reported that a 12-week multimodal intervention combining exercise and dietary guidance produced a ~2.2% deceleration in DunedinPACE relative to controls [142]. However, the magnitude and clinical significance of changes in epigenetic clocks may depend on intervention duration, population characteristics and the specific biomarker analysed.

**Figure 5:**
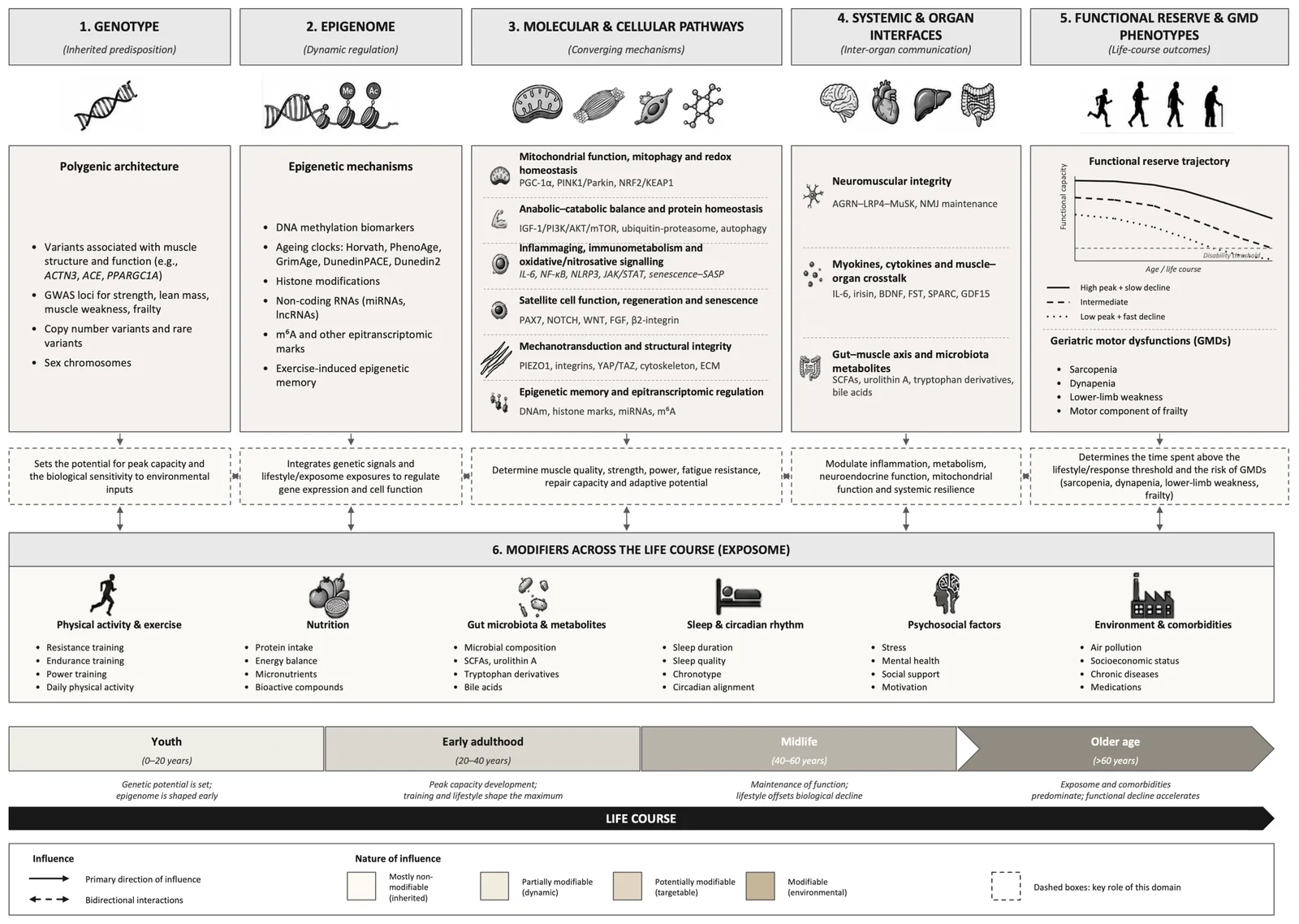
Integrated life-course framework linking genetic architecture, epigenomic regulation, biological pathways and functional reserve. The model illustrates how inherited genetic predisposition, including candidate genes, genome-wide association study loci and polygenic architecture, interacts with dynamic epigenomic regulation to influence converging molecular and cellular pathways. These mechanisms, in turn, shape systemic and organ-level communication involving the neuromuscular system, myokines and exerkines, and the gut–muscle axis. Their combined effects determine the attainment and maintenance of functional reserve and influence vulnerability to geriatric motor dysfunctions, including sarcopenia, dynapenia, lower-limb weakness and the motor component of frailty. Physical activity, nutrition, gut microbiota, sleep and circadian rhythms, psychosocial factors, environmental exposures and comorbidities act as modifiers throughout the life course. Solid arrows indicate the principal direction of influence, whereas bidirectional arrows represent reciprocal interactions between biological domains and life-course exposures.

Taken together, these data support a view in which epigenetic clocks may constitute a translational bridge between the molecular-level pathways described earlier in this section and the whole-person phenotype of functional reserve (see Figure 5). As reviewed by Ibáñez-Cabellos and colleagues, the next generation of epigenetic clocks (simpler, cheaper and better validated across diverse populations) is likely to become a routine tool for individualised monitoring of biological ageing and for evaluating the effectiveness of exercise and nutritional interventions targeting GMDs [41].

## 6. Gut Microbiota: The Modulatory Layer of the Gut–Muscle Axis

The gut microbiota has a direct impact on metabolic, inflammatory and neurological processes, as well as on muscle function. The gut–muscle axis has gained prominence following observations that microbial composition affects both muscle mass and function in older adults, as well as their response to training [143,144]. Although this field is still under development, it highlights a key point: not all the biological factors that influence muscle function are directly encoded in the human genome.

Microbial metabolites are the main focus of interest. Short-chain fatty acids (SCFAs), particularly butyrate, modulate inflammation, insulin sensitivity, the integrity of the intestinal barrier and gene expression, amongst other factors. In old age, a less diverse microbiota, with a loss of butyrate-producing bacteria and an increase in pro-inflammatory signals, could contribute to the onset of inflammageing and anabolic resistance. Furthermore, in the context of sport, a more diverse microbiota that is functionally oriented towards energy metabolism could promote recovery and the availability of substrates.

Urolithin A is a prime example of the interaction between diet, the gut microbiota and mitochondrial function. This metabolite, produced by the gut microbiota from dietary ellagitannins, has been linked to the activation of mitophagy and improvements in mitochondrial biomarkers in humans [145]. The randomised clinical trial by Liu et al. in adults aged 65 and over showed that supplementation with 1000 mg/day for 4 months significantly improved muscle endurance (assessed by the number of knee extension repetitions performed) and biomarkers of mitochondrial health [146]. Beyond the significance of this specific finding, urolithin A demonstrates how a biological pathway can be modulated by mechanisms that depend on the host, diet and gut ecology.

The comparison between athletes and sedentary individuals reinforces the impact of the microbiome on the body. Studies in athletes have described greater microbial diversity and an abundance of short-chain fatty acid producers, alongside distinct functional profiles in terms of energy metabolism [147,148]. Using metabolomics, Scheiman and colleagues identified a higher abundance of *Veillonella atypica*, a bacterium capable of converting lactate into propionate, in Olympic marathon runners, providing experimental evidence of a microbial metabolite with an ergogenic effect [149]. Exercise interventions in sedentary individuals have also shown that physical activity can reversibly alter the composition and function of the gut microbiota [150].

Genetic predisposition does not, therefore, operate in a vacuum: the microbiota modulates pathways that are also relevant to performance or metabolic health, such as inflammation, energy metabolism and mitochondrial function. The gut–muscle axis broadens the genetic framework and makes it more dependent on the life course and lifestyle.

Recent studies have shed light on the causal architecture of the gut–muscle axis. A large-scale Mendelian-randomisation study by Zhang and colleagues identified specific microbial taxa and metabolites, including tryptophan derivatives and short-chain fatty acids, as causally associated with sarcopenia-related traits, providing genetic support for the observational associations described above [151]. Xu and colleagues have demonstrated that gut-microbiota-mediated tryptophan metabolism modulates inflammageing in frailty and sarcopenia, closing the mechanistic loop between microbiota, immunometabolism and functional decline [152]. Systematic reviews indicate that exercise interventions may modestly increase gut-microbial diversity and alter microbial composition, although responses vary according to age, sex, baseline characteristics and intervention design [153], and dedicated reviews of the gut–muscle axis have proposed exercise as an integrative intervention that simultaneously modulates microbial composition, muscle protein turnover and systemic inflammation [154]. Taken together, these advances position the gut microbiota as a modifiable interface between the exposome and the genetic architecture of metabolic disorders, while supporting the development of microbiota-targeted adjunctive interventions to complement physical exercise.

## 7. Pleiotropy, Trade-Offs and the Exposome: How to Interpret the Relationship Between Performance and GMDs

Pleiotropy explains why a single variant or biological pathway can influence multiple phenotypes. Favourable pleiotropy would be the most intuitive scenario: variants that promote greater mitochondrial efficiency, a better response to training, adequate anabolic signalling or reduced chronic inflammation could be associated with both improved physical performance and a lower risk of developing GMDs. Under this interpretation, athletic performance would not be at odds with functional health, but rather an early expression of robust biological systems. The Mendelian randomisation of the GWAS by Jones et al. supports a causal link between muscle weakness and frailty [26].

*ACTN3* partly illustrates this logic, although it also highlights its limitations. The genotype associated with power may favour certain sporting demands in young people [43], but its isolated effect is unlikely to predict a person’s muscle ageing. Something similar applies to *ACE* or *PPARGC1A*: these are biologically plausible genes that are useful for understanding mechanisms, but they do not, on their own, determine a person’s life course. Pleiotropy must be interpreted at the level of networks and pathways, not as a simple relationship between a variant and a clinical outcome.

Antagonistic pleiotropy, Williams’ evolutionary principle that biological traits or alleles conferring advantages earlier in life may exert adverse effects at post-reproductive ages, introduces a more complex interpretation of the relationship between performance and ageing. Several biological axes relevant to this review illustrate the concept in different ways:

First, the IGF-1/insulin/mTOR axis constitutes the paradigmatic example. Elevated IGF-1 signalling promotes muscle protein synthesis, hypertrophy and neuromuscular development during growth and adulthood, but sustained activation is associated with reduced longevity in model organisms and with increased risk of some age-related diseases in humans, as reviewed by Zhang and colleagues [155]. Recent bidirectional Mendelian-randomisation analyses have refined this picture, showing that genetically instrumented components of the IGF family exert context-dependent causal effects on muscle mass and strength [156]. Rather than a simple trade-off, IGF-1 signalling exhibits a “sweet spot”: chronic hyperactivation may accelerate senescence pathways, whereas insufficient signalling accelerates anabolic resistance and sarcopenia. Exercise appears to modulate this axis toward the beneficial end of the curve.

Second, telomere biology provides an evolutionary example of a shared limiting resource. Longer telomeres and stronger telomerase activity favour tissue regeneration (including satellite cell function and cardiovascular health) but excessive telomerase activity is associated with proliferative risk. A recent bidirectional Mendelian-randomisation analysis has identified a potential causal relationship between leukocyte telomere length and sarcopenia-related traits, whereby shorter telomeres may contribute to lower muscle mass and strength, while impaired muscle function may in turn be associated with accelerated telomere attrition [157]. Endurance training has been associated with the maintenance of telomere length in older adults, offering a plausible non-pharmacological approach to buffer this trade-off [158].

Third, redox-inflammatory signalling embodies a hormetic trade-off. As discussed in previous sections, acute exercise-induced reactive oxygen species and transient IL-6 elevations are essential for training adaptation, but chronic activation of the same pathways drives inflammageing and frailty.

Fourth, mTOR remains the canonical intracellular integrator of these trade-offs. mTORC1 activation is indispensable for muscle protein synthesis and hypertrophy, yet its chronic activation suppresses autophagy and accelerates cellular ageing; caloric restriction, rapamycin analogues and endurance exercise all converge on partial mTOR modulation to attenuate this cost.

Direct human evidence that specific performance-enhancing variants increase late-life GMD risk remains limited: the *ACTN3* XX genotype, for example, has not been convincingly associated with accelerated sarcopenia or frailty in longitudinal cohorts, and the LASA data suggest that the phenotypic effect of grip-strength PRS on frailty is largely mediated by environmental factors [55]. However, the current literature on IGF-1 [156], telomeres [157], and inflammation supports the possibility that pleiotropic effects, though modest at the level of each individual variant, may cumulatively determine the trajectory of functional reserve over the course of a lifetime.

The exposome, defined as the totality of environmental exposures across the life course, together with the biological responses they elicit, provides the operational counterpart to genetic predisposition in the life-course model. Concrete examples illustrate how these exposures may modify functional trajectories. Structured leisure-time exercise is consistently associated with healthier ageing, whereas prolonged occupational loading performed with limited recovery may not confer the same health benefits as structured leisure-time exercise, a phenomenon described as the physical activity paradox [159]. Socioeconomic circumstances influence access to adequate nutrition, safe environments for physical activity and healthcare and may therefore modify the expression of genetic predisposition to muscle strength and functional decline [65,160]. Ambient air pollution is emerging as a potent exposome factor: recent Chinese national data show that improvements in air quality are associated with slower frailty progression [161], and community-based cohorts report significant associations between long-term PM2.5 exposure and both sarcopenia and cognitive decline [162]. Indoor air pollution generated by solid-fuel combustion for cooking and heating has been longitudinally linked to sarcopenia onset and progression, with a particularly strong effect in middle-aged women [163]. Features of the built environment, sleep quality, psychosocial stress and circadian disruption may further influence habitual activity, metabolic health and redox-inflammatory regulation [164,165]. Finally, the Pandics and colleagues review of environmental drivers of unhealthy ageing highlights the cumulative and interactive nature of these exposures [166]. The exposome therefore provides both a biological explanation for gene–environment interactions and a set of potentially modifiable targets for clinical and public-health intervention.

## 8. Empirical Evidence: Longitudinal Trajectories and Common Markers

Studies have been conducted linking objective physical capacity to mortality, showing that strength, speed and functional performance are not merely sporting or geriatric measures, but indicators of systemic health [30]. Grip strength follows typical patterns throughout the life course and is associated with adverse outcomes when it falls below the expected values for a given age and sex [167]. The LASA study mentioned above confirms precisely that grip strength predicts frailty and functional limitation, illustrating the significance of the exposome [55].

A person’s sporting history and sustained physical activity over time are also significant. Former athletes and people who have maintained high levels of activity tend to have better physical function in later life, although these studies should be interpreted with caution due to potential selection bias: those who went on to become elite athletes may already have had genetic, social or health-related advantages. Master athletes provide a particularly useful analytical framework by distinguishing intrinsic ageing from factors attributable to inactivity or chronic disease [168]; however, regardless of this, the practical message remains the same: training for strength, power, balance and aerobic capacity improves function in older adults and can partially reverse factors attributable to sarcopenia or frailty [1,2].

Furthermore, functional markers provide a common language. Grip strength summarises overall strength levels and is associated with prognosis; walking speed incorporates lower-limb power, balance, neuromotor control and cardiorespiratory fitness; the SPPB combines balance, walking and the ability to stand up from a chair, and therefore predicts mortality and disability [55,168]. These tools are valuable because they enable complex molecular mechanisms to be translated into observable clinical outcomes.

At the genetic level, GWAS and PRS are beginning to enable cross-analyses between performance traits, body composition and physical function [25,26,50,51,52]. There is still a lack of studies directly linking genotypes associated with athletic performance in youth to diagnoses of sarcopenia or frailty decades later. This absence does not invalidate the hypothesis, but it does mean that it must be framed as a research programme rather than a definitive conclusion.

The microbiota provides a second line of complementary evidence. Differences between athletes and sedentary individuals [147,148], findings on microorganisms involved in lactate metabolism [149] and the ability of exercise to alter the microbiota [150] suggest that physical activity reshapes not only muscle but also associated systems. In older adults, the relationship between the microbiota, muscle mass and frailty [143] points in the same direction: functional decline may depend on biological networks that go beyond the muscle as an isolated organ.

## 9. Practical Implications: Preserving Functional Reserve Through Exercise, Early Screening and Risk Communication

The proposed framework has practical implications, but it should also be interpreted with caution. In research, integrating genetics, physical function, inflammatory biomarkers, body composition, the microbiota and longitudinal follow-up can help to identify risk subgroups and dominant mechanisms. In current clinical practice, however, the most useful tools remain the simplest: grip strength, walking speed, SPPB, history of falls, level of physical activity, nutritional status and presence of comorbidities.

From a clinical perspective, sport and exercise should not be viewed solely as tools for improving performance, but also as preventive measures against GMDs. Sports activities and programmes tailored to an individual’s specific circumstances can incorporate aerobic training, strength, power, balance, coordination and social engagement, all of which are important for maintaining functional reserve. International physical activity guidelines are clear on this point, recognising the benefits for adults and older adults in terms of mortality, cardiovascular disease, type 2 diabetes, mental health, cognitive function, falls and body composition. Furthermore, clinical trials such as the LIFE Study have shown that a structured physical activity programme can reduce the onset of major mobility disability in older adults at functional risk [169]. Therefore, sport adapted to age, clinical condition and functional level can be considered a relatively low-cost clinical strategy with high potential impact for delaying the transition from preserved function to sarcopenia, frailty or lower-limb weakness.

Furthermore, it is important to emphasise that it is never too early or too late to start or maintain a physically active lifestyle. In early life, physical activity and training help to maximise peak functional capacity, increasing the reserve capacity against subsequent decline. In older age, even when there is already some loss of strength, mobility or balance, exercise is still capable of inducing significant adaptations. The World Health Organization’s guidelines highlight that any amount of physical activity is better than none, that all activity counts, and that muscle strengthening brings benefits across all age groups; this is particularly important given that inactivity increases from the age of 60 onwards, precisely at the stage when maintaining strength, power and balance becomes most crucial for preventing falls, dependency or loss of independence [170].

The preventive benefits of exercise must also be understood in light of the role of skeletal muscle as an endocrine organ, as mentioned above. Muscle contraction triggers the release of myokines and other secreted factors capable of modulating systemic metabolism, inflammation, vascular function, bone homeostasis, muscle–brain communication and adaptation to physiological stress [117,118]. The loss of muscle mass and function represents not only a mechanical limitation but also a reduction in the body’s endocrine and metabolic capacity. Therefore, interventions such as strength, power and endurance training can act simultaneously on the muscle as both contractile tissue and a secretory organ, promoting a systemic profile more compatible with functional resilience and reinforcing the need to consider sarcopenia and frailty as multisystemic syndromes, not merely as localised problems at the level of muscle mass [119,120].

Furthermore, in a context where life expectancy continues to rise, the functional tests used in clinical practice should be adapted to a more ambitious goal: not only to identify established disability, but also to detect early losses of intrinsic capacity and functional reserve. Classic tests such as handgrip strength, walking speed, the SPPB, the 5STS or the Timed Up and Go (TUG) remain useful due to their low cost, reproducibility and prognostic value. However, in the context of an ageing population, these measures should be complemented by a more dynamic and longitudinal interpretation, based on individual trajectories, intra-individual changes and the early detection of subtle declines. The WHO has already proposed a model of integrated care designed to guide healthcare systems towards optimising intrinsic capacity and functional ability, rather than limiting themselves to the late diagnosis of a disease or disability [171].

Genetic screening could prove useful for risk stratification before symptoms appear, particularly when combined with functional and environmental data. However, the available PRSs have significant limitations: moderate predictive power, insufficient validation in non-European populations, and unestablished incremental utility compared to inexpensive and reproducible functional tests [55]. For this reason, their use should, for the time being, be confined to research or very specific contexts, and not serve as a substitute for clinical assessment.

Preventive interventions should prioritise components that are broadly beneficial. Strength training is key to maintaining muscle mass, strength and function [2]; strength, power and balance training help to reduce the risk of falls; aerobic exercise preserves cardiorespiratory capacity and oxidative metabolism; and nutrition should ensure optimal protein synthesis, while supporting metabolic health and limiting chronic low-grade inflammatory burden. In this regard, dietary patterns rich in anti-inflammatory and redox-active bioactive compounds, including polyphenols found in berries, green tea and extra virgin olive oil, carotenoids and vitamins C and E, may contribute to a more favourable inflammatory and oxidative profile, although they should be interpreted as part of an integrated lifestyle strategy rather than as isolated antioxidant interventions [19,172,173,174,175,176]. Some emerging interventions based on microbial metabolites, such as urolithin A, are expanding the therapeutic repertoire by targeting mitochondrial quality control and potentially modulating redox-inflammatory pathways. These compounds provide a useful example of how diet–microbiota interactions may influence muscle ageing; however, their long-term clinical relevance for the preservation of muscle function remains to be established [146]. Overall, although genetics may influence the magnitude of the response, mechanical, metabolic and nutritional stimuli remain necessary.

Finally, it is worth noting that communicating genetic risk requires particular care: presenting a variant or a score as an inevitable fate can lead to a sense of fatalism, anxiety or even stigmatisation. The correct framing is that of a modifiable predisposition: genetics may shift the starting point or sensitivity to certain stimuli, but it does not negate the effect of exercise, nutrition or disease management. This distinction is ethically and clinically essential, because the aim should not be to classify individuals as predestined to develop a GMD, but rather to identify early opportunities for prevention.

## 10. Limitations and Research Agenda

The main limitation in this field is phenotypic heterogeneity: Firstly, athletic performance encompasses very different disciplines, such as endurance, power, technical sports, team sports, combined events, etc., whilst sarcopenia and frailty vary according to the definitions provided by the various consensus groups (EWGSOP2, AWGS2, SDOC or FNIH), cut-off points and populations [37,39]. This heterogeneity makes it difficult to compare studies and may explain some of the inconsistencies observed in the genetics of performance.

A second limitation is the lack of life-course cohorts with in-depth functional phenotyping: many biobanks have genotypes and large sample sizes, but do not always include detailed measures of strength, muscle composition, sporting history, training or neuromuscular function, amongst other factors. Conversely, studies with more comprehensive functional testing tend to have fewer participants or shorter follow-up periods. To robustly test the shared architecture hypothesis, cohorts are needed that span youth, middle age and old age, with repeated measurements of performance, strength, gait, sarcopenia, frailty, physical activity, nutrition, microbiota or comorbidities.

Population transferability is another challenge: a large proportion of genetic studies have been conducted in populations of European descent, which limits the application of PRS to other groups. Furthermore, muscle mass, strength or the rate of decline vary according to sex, age, hormonal status or sociocultural context; therefore, the development of precision medicine for muscle function will require more diverse studies and stratified analyses that do not obscure certain relevant interactions.

Finally, causality remains difficult to establish. The fact that two phenotypes share genetic pathways or statistical correlations does not imply that a variant associated with improved performance directly protects against a GMD. Longitudinal analyses, Mendelian-randomisation studies, controlled interventions and mechanistic models are required to distinguish the effects of genetic predisposition, athletic selection, cumulative training, health behaviour and environmental exposure [25,26]. The growing availability of genomic, transcriptomic, epigenomic, metabolomic and microbiome data in longitudinal cohorts offers an important opportunity to integrate these layers into predictive models of functional trajectories.

## 11. Conclusions: From the Genetics of Performance to Precision Approaches for Functional Ageing

Athletic performance and age-related motor decline can be interpreted within a shared life-course framework. The genetic and biological mechanisms influencing strength, power, endurance and trainability remain relevant in later life, but are expressed within a physiological context increasingly shaped by declining reserve, comorbidities, inactivity and cumulative environmental exposure.

The available evidence supports partial convergence across mitochondrial function and mitophagy, anabolic–catabolic balance, neuromuscular integrity, satellite cell reserve, mechanotransduction, myokine- and exerkine-mediated communication, epigenetic regulation and the gut–muscle axis. Redox and inflammatory signalling represent an important integrative component of these networks: transient activation contributes to adaptation and repair, whereas chronic dysregulation is associated with impaired regeneration and functional decline. Much of this relationship is compatible with favourable pleiotropy, although antagonistic and context-dependent effects remain plausible and require more direct evaluation in longitudinal human studies.

The practical message is that genetic predisposition influences, but does not determine, functional ageing. Functional reserve can be supported by maximising peak capacity in early life and slowing functional decline throughout adulthood and older age. Exercise, particularly resistance, power, balance and aerobic training, together with adequate nutrition and effective management of comorbidities, remains one of the most effective and broadly accessible strategies for preserving mobility and independence. Future approaches may integrate genetic information, longitudinal functional phenotypes, biological-age indicators and exposome data to improve risk stratification and intervention monitoring. However, these tools should complement rather than replace direct functional assessment, and their clinical utility requires validation in diverse populations.

Ultimately, the value of this framework lies not in identifying individuals who are genetically destined to perform well or age poorly, but in clarifying how biological predisposition and modifiable exposures interact to shape functional reserve. Preserving autonomy, mobility and quality of life should remain the principal clinical objective.

## Data Availability

No new data were created or analyzed in this study. Data sharing is not applicable to this article.

## Abbreviations

1-RM	One-repetition maximum
5STS	Five Times Sit-to-Stand Test
*ACE*	Angiotensin-converting enzyme
*ACTN3*	Alpha-actinin-3
ADLs	Activities of daily living
AMPK	AMP-activated protein kinase
ATP	Adenosine triphosphate
AWGS2	Asian Working Group for Sarcopenia 2
CARDIA	Coronary Artery Risk Development in Young Adults
DNA	Deoxyribonucleic acid
DXA	Dual-energy X-ray absorptiometry
ECM	Extracellular matrix
EWGSOP2	European Working Group on Sarcopenia in Older People 2
FNIH	Foundation for the National Institutes of Health
*FTO*	Fat mass and obesity-associated gene
GMD	Geriatric motor dysfunction
GMDs	Geriatric motor dysfunctions
GWAS	Genome-wide association studies
HERITAGE	Health, Risk Factors, Exercise Training and Genetics Family Study
I/D	Insertion/deletion
ICOPE	Integrated care for older people
IGF-1	Insulin-like growth factor 1
IL-6	Interleukin-6
LASA	Longitudinal Aging Study Amsterdam
LIFE	Lifestyle Interventions and Independence for Elders
m^6^A	N6-methyladenosine
MDPs	Mitochondria-derived peptides
miRNAs	MicroRNAs
MOTS-c	Mitochondrial open reading frame of the 12S rRNA-c
MR	Mendelian randomisation
mTOR	Mechanistic target of rapamycin
mTORC1	Mechanistic target of rapamycin complex 1
mtDNA	Mitochondrial DNA
NMJ	Neuromuscular junction
PGC-1α	Peroxisome proliferator-activated receptor gamma coactivator 1-alpha
PI3K	Phosphoinositide 3-kinase
*PINK1*	PTEN-induced kinase 1
PPAR	Peroxisome proliferator-activated receptor
*PPARGC1A*	Gene encoding PGC-1α
PRS	Polygenic risk score
SCFAs	Short-chain fatty acids
SDOC	Sarcopenia Definition and Outcomes Consortium
SHLPs	Small humanin-like peptides
SNP	Single-nucleotide polymorphism
SPPB	Short Physical Performance Battery
TGF-β	Transforming growth factor beta
TNF-α	Tumour necrosis factor alpha
TUG	Timed Up and Go
VO_2max_	Maximal oxygen uptake
WHO	World Health Organization
